# Simultaneous valorization and biocatalytic upgrading of heavy vacuum gas oil by the biosurfactant‐producing *Pseudomonas aeruginosa* AK6U

**DOI:** 10.1111/1751-7915.12741

**Published:** 2017-07-11

**Authors:** Wael Ahmed Ismail, Magdy El‐Said Mohamed, Maysoon N. Awadh, Christian Obuekwe, Ashraf M. El Nayal

**Affiliations:** ^1^ Environmental Biotechnology Program Life Sciences Department College of Graduate Studies Arabian Gulf University Manama Kingdom of Bahrain; ^2^ Biotechnology, Research and Development Center Saudi Aramco Dhahran Saudi Arabia; ^3^ Department of Biological Sciences College of Science Kuwait University Kuwait Kuwait

## Abstract

Heavy vacuum gas oil (HVGO) is a complex and viscous hydrocarbon stream that is produced as the bottom side product from the vacuum distillation units in petroleum refineries. HVGO is conventionally treated with thermochemical process, which is costly and environmentally polluting. Here, we investigate two petroleum biotechnology applications, namely valorization and bioupgrading, as green approaches for valorization and upgrading of HVGO. The *Pseudomonas aeruginosa* AK6U strain grew on 20% v/v of HVGO as a sole carbon and sulfur source. It produced rhamnolipid biosurfactants in a growth‐associated mode with a maximum crude biosurfactants yield of 10.1 g l^−1^, which reduced the surface tension of the cell‐free culture supernatant to 30.6 mN m^−1^ within 1 week of incubation. The rarely occurring dirhamnolipid Rha–Rha–C_12_–C_12_ dominated the congeners’ profile of the biosurfactants produced from HVGO. Heavy vacuum gas oil was recovered from the cultures and abiotic controls and the maltene fraction was extracted for further analysis. Fractional distillation (SimDist) of the biotreated maltene fraction showed a relative decrease in the high‐boiling heavy fuel fraction (BP 426–565 °C) concomitant with increase in the lighter distillate diesel fraction (BP 315–426 °C). Analysis of the maltene fraction revealed compositional changes. The number‐average (Mn) and weight‐average (Mw) molecular weights, as well as the absolute number of hydrocarbons and sulfur heterocycles were higher in the biotreated maltene fraction of HVGO. These findings suggest that HVGO can be potentially exploited as a carbon‐rich substrate for production of the high‐value biosurfactants by *P. aeruginosa* AK6U and to concomitantly improve/upgrade its chemical composition.

## Introduction

Biosurfactants are surface‐active microbial products that are getting increasing interest due to their superior physicochemical properties, environmental compatibility as compared with synthetic (petroleum‐based) surfactants (Soberón‐Chávez and Maier, 2011), and the wide range of industrial and environmental applications. In the petrochemical industry, they can be applied in enhanced oil recovery, oil spill cleaning, tanker cleanup, viscosity control, emulsification and formulation of petrochemicals (De Almeida *et al*., 2016). The biosurfactants market share is projected to reach more than two billion US $ by 2020 with the production of ca 462 kilo tons (De Almeida *et al*., 2016).

Despite extensive efforts, biosurfactants have not been able to compete economically with their synthetic counterparts due to high production costs. To overcome this problem, at least in part, low‐value and inexpensive carbon‐rich feed stocks, such as agricultural and industrial wastes/residues, have been used by many investigators as carbon sources for biosurfactants production (Banat *et al*., 2014). The petroleum industry also generates huge amounts of wastes and residues that represent an environmental burden including refinery wastes, sludge, wastewater and difficult‐to‐refine hydrocarbon streams (Gray, 1994). Currently, ca 725 million metric tons of residue, resulting from the vacuum distillation units, is processed through various conversion processes (Sahu *et al*., 2015). Although petrochemical wastes and processing residues are potential substrates for biosurfactants production, they have not received the proper attention.

**Figure 1:**
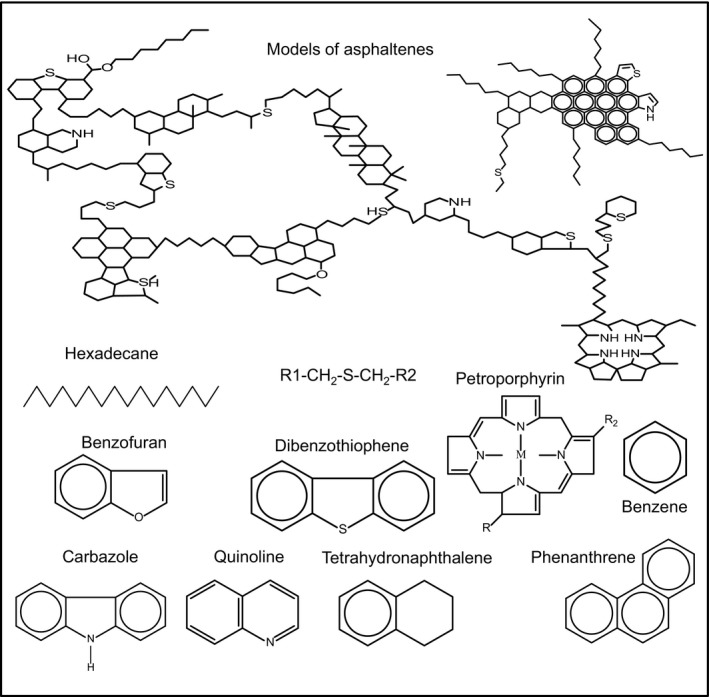
Chemical structures of representative hydrocarbon components of HVGO.

Initial processing of crude petroleum oil in the atmospheric distillation units produces a heavy residue (atmospheric residue), which is then further fractionated in the vacuum distillation tower into heavy vacuum gas oil (HVGO) and vacuum residue (VR). HVGO (intermediate product) is the bottom side product (boiling range 398–565 °C) from the vacuum distillation tower, whereas VR is the bottom product (boiling range 565 °C+; Gray, 1994; Ramirez‐Corredores and Borole, 2006; Sahu *et al*., 2015). HVGO and VR are viscous, heavy and contain high molecular weight and structurally complex components such as asphaltenes and maltenes (resins, saturates, aromatics) in addition to metals (Ni and V) and heteroatoms (N and S) in different proportions (Ramirez‐Corredores and Borole, 2006; Sahu *et al*., 2015; Fig. 1). Asphaltenes constitute the highest molecular weight and most polar fraction of the crude oil. It consists of polycyclic aromatic hydrocarbons interlinked with side‐chains, sulfur and nitrogen containing heterocycles, metal‐containing petroporphyrins and polar functional groups (Gray, 1994; Pineda‐Flores and Mesta‐Howard, 2001; Ramirez‐Corredores and Borole, 2006). The maltene fraction comprises resins (smaller versions of asphaltenes), aromatic compounds, as well as straight, branched and alicyclic hydrocarbons (Gray, 1994; Speight, 2013; Fig. 1).

These physicochemical characteristics reduce the economic profit of HVGO and VR due to the challenges associated with their refining/processing. So, how are they conventionally processed in oil refineries? Conversion of HVGO and VR into high‐value distillable oils/fuels implies upgrading pretreatments via thermochemical processes (hydrotreatment and hydrocracking; Gray, 1994; Ramirez‐Corredores and Borole, 2006). The ultimate goal of the upgrading treatment is viscosity reduction and conversion of these low‐value fractions into higher value products that can be distilled into fuels such as diesel and gasoline. This can be achieved by converting the high‐boiling complex structures (asphaltenes and resins) into low‐boiling smaller molecular weight components and removing undesirable impurities (metals and heteroatoms; Ramirez‐Corredores and Borole, 2006; Mazaheri and Tabatabaee, 2010; Sahu *et al*., 2015). The conventional upgrading treatments are costly and environmentally polluting (Le Borgne and Quintero, 2003). They operate under sever conditions of temperature (up to 480 °C) and pressure (up to 4000 psi) and require huge amounts of catalysts (Gray, 1994; Ramirez‐Corredores and Borole, 2006). The severity and, consequently, the cost of the upgrading processes are expected to increase because of the inevitable change of the refinery feed to heavy crude oil and bitumen. Those unconventional crude oil resources contain higher amounts of the problematic components (asphaltenes, metals and heteroatoms), and therefore, their refining produces heavier cuts and residues (Ramirez‐Corredores and Borole, 2006; Speight, 2013).

As compared with conventional physicochemical techniques, biotechnological processes are environmentally compatible, cost‐effective and endowed with high selectivity (Le Borgne and Quintero, 2003; Kilbane, 2006). Therefore, enhancing the physicochemical characteristics of HVGO and other refining residues via biotechnology‐based bioupgrading processes is worth investigating. Moreover, bioconversion of HVGO, as a carbon source, to value‐added biosurfactants is an appealing valorization approach. This is because HVGO is carbon‐rich and difficult‐to‐refine hydrocarbon stream due to challenging physicochemical properties (Ramirez‐Corredores and Borole, 2006; Sahu *et al*., 2015). The literature lacks, to our knowledge, any studies on HVGO bioupgrading or bioconversion to biosurfactants. We found only a few reports on the biotransformation of asphaltene fractions extracted from crude oil. The majority of those investigations focused on lignin‐degrading fungi and their extracellular oxidative enzymes such as peroxidases and laccases (Fedorak *et al*., 1993; Garcia‐Arellano *et al*., 2004; Naranjo *et al*., 2007; Uribe‐Alvarez *et al*., 2011; Ayala *et al*., 2012). Nonetheless, results of asphaltenes biodegradation/biotransformation were mostly circumstantial and could be due to abiotic losses or modification. Moreover, some asphaltenes biotransformation experiments contained other carbon sources in addition to asphaltenes, which might make the results misleading (Pineda‐Flores *et al*., 2004; Uribe‐Alvarez *et al*., 2011). Recently, Hernández‐López *et al*. (2015) investigated the differential gene expression in *Neosartorya fischeri* grown on either petroleum asphaltenes or glucose–peptone. Genes encoding aromatic hydrocarbon monooxygenases were among those upregulated in asphaltenes‐grown cells.

In this study, we addressed two questions. The first: Can bacteria utilize HVGO as a carbon source for biosurfactants production? The second: What is the effect of bacterial growth on the chemical composition of HVGO? To answer these questions, we performed bioconversion and bioupgrading experiments on HVGO with a biosurfactant‐producing *Pseudomonas aeruginosa* AK6U strain, which was recently isolated and characterised in our laboratory (Ismail *et al*., 2014, 2015).

## Results and discussion

### Growth of the AK6U strain on HVGO and biosurfactants production

**Figure 2:**
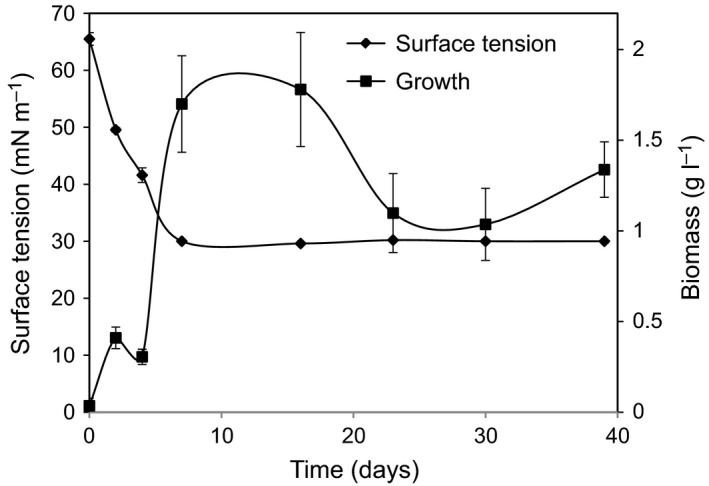
A graph showing the changes in biomass formation and biosurfactants production (shown as decrease in surface tension) during growth of *P. aeruginosa* AK6U on HVGO (20% v/v) as the sole carbon and sulfur source in mineral salts medium.

To investigate the ability of the *P. aeruginosa* AK6U strain to grow on HVGO and produce biosurfactants, we cultured the strain in sulfur‐free mineral salts medium (MSM) containing HVGO as a sole carbon and sulfur source. The AK6U strain grew in the biphasic cultures containing 20% (v/v) of HVGO. Visual inspection of the cultures revealed temporal changes in colour and turbidity of the cultures as well as consistency of the oil (Fig. S1), as compared with the uninoculated controls. Towards the end of the incubation period, it was not possible to distinguish the HVGO layer from the aqueous phase (MSM), and the cultures started to develop foaming. Growth of AK6U was confirmed by measuring the temporal changes in the biomass dry weight as shown in Fig. 2. In the presence of 20% HVGO, the growth increased, and the cultures attained a maximum biomass of 1.8 ± 0.29 g after 16 days of incubation. Then the biomass declined and became stable after the 22nd day of incubation until the end of the experiment. HVGO, as described in the introduction, is a carbon‐rich substrate. Accordingly, growth of AK6U on HVGO shows that it has utilized the oil as a carbon source for energy production and biomass formation. Furthermore, as HVGO was the sole sulfur source in the culture, we assume that the AK6U strain satisfied its sulfur requirements from the sulfur content of HVGO. The latter is known to contain organosulfur compounds like dibenzothiophene and its alkylated derivatives (Gray, 1994; Ramirez‐Corredores and Borole, 2006). In line with this, we have shown in previous studies that AK6U can utilize several organosulfur compounds such as dibenzothiophene, benzothiophene, 4‐methyldibenzothiophene, 4,6‐dimethyldibenzothiophene and dibenzylsulfide as sole sulfur sources (Ismail *et al*., 2014, 2015).

Although the graph in Fig. 2 shows the common growth profile in terms of growth phases, we postulate that it does not represent the actual growth curve, particularly in terms of the length of the phases and the biomass yield. This assumption is based on the observation that large biomass aggregates were attached to oil‐growth medium interphase and the sides of the centrifuge tubes (Fig. S2). We have seen this during final centrifugation at the end of the incubation period to collect cell‐free culture supernatants. At this time, only very small cell pellet could be collected at the bottom of the centrifuge tubes. To quantify the biomass for the growth curve, we depended on culture samples taken from the aqueous phase (no oil). This means, we were quantifying biomass suspended in the aqueous phase, not that which was associated with HVGO. Adherence of bacterial cells to hydrophobic substrates like oil is well documented in the literature. Many oil‐ and hydrocarbon‐degrading bacteria have hydrophobic cell surface that enables attachment to water‐insoluble substrates (Bredholt *et al*., 1998; Obuekwe *et al*., 2009). Some bacteria, like *Pseudomonas* spp., develop hydrophobic cell surface when challenged with hydrophobic substrates (Warne *et al*., 2010; Feng *et al*., 2013). This is one strategy to get access to the otherwise non‐accessible or hardly accessible hydrophobic compounds. The ultimate goal is to enhance bioavailability and, consequently, biodegradability.

To gain a direct evidence for biosurfactants production, we followed the change in surface tension of the cultures with time. As shown in Fig. 2, the surface tension decreased with time and reached a minimum of 30.6 mN m^−1^ after 7 days of incubation in the presence of 20% HVGO. No further reduction in surface tension occurred after 7 days until the end of measurement period (39 days). Reduction in the surface tension confirms that AK6U utilized HVGO as a carbon and sulfur source for growth and biosurfactants production (Soberón‐Chávez and Maier, 2011). Furthermore, as the reduction of surface tension was measured in cell‐free culture supernatants, it can be concluded that the biosurfactants produced from HVGO were excreted into the extracellular medium. The kinetics of biosurfactants production showed a growth‐associated mode. After 18 days of incubation with 20% HVGO, we recovered 9.8 g l^−1^ of crude biosurfactants from cell‐free culture supernatants. This yield increased a little to 10.1 g l^−1^ after 39 days.

Production of biosurfactants by some bacteria, including many *Pseudomonas* spp., when challenged with hydrophobic substrates such as HVGO is a common physiological adaptation. Biosurfactants enhance the bioavailability of water‐insoluble or water‐immiscible hydrocarbons. Thus, enabling the bacterial cells to better access those substrates, which consequently promotes biodegradation efficiency (Ward, 2010; Mnif *et al*., 2011). The detection of biosurfactants in cell‐free culture supernatants is an interesting feature from an industrial perspective and points to a potential role of biosurfactants in the utilization of HVGO, which exists extracellularly. This is also coherent with the observed growth‐associated decrease in surface tension during the initial days of incubation.

As mentioned under the methodology, the culture medium was sulfur‐free, and HVGO was provided as the sole sulfur source. Actually, this was done on purpose. We know from our previous studies that biosurfactants production by the AK6U strain is stimulated when the conventional sulfur source (inorganic sulfate) is replaced by organosulfur compounds (Ismail *et al*., 2014, 2015). Therefore, we excluded inorganic sulfate from the culture medium to force the strain to utilize the organosulfur fraction from HVGO as a sulfur source and promote biosurfactants production. It appears that this has worked as the strain grew very well, which is in line with the reported capacity of AK6U to utilize several organosulfur compounds commonly found in crude oil and refined products as sulfur sources (Gray, 1994; Ramirez‐Corredores and Borole, 2006; Ismail *et al*., 2015).

It also appears that production of biosurfactants occurred mainly during the first 2 weeks of incubation. This is because there was no remarkable increase in biosurfactants yield after the 18th day of incubation until the 39th day. The highest yield was 10.1 g l^−1^. This is twofold to sixfold higher than the value reported for other *P. aeruginosa* strains using glycerol and vegetable oils as carbon sources (Rahman *et al*., 2002; Kumar *et al*., 2012). This yield is also much higher (2.8‐fold to 20‐fold) than yields reported in previous studies for the AK6U strain using glycerol, glucose or vegetable oils as carbon sources (AlKhalifa, 2013; Ismail *et al*., 2014, 2015). The variation in the biosurfactants yield is in line with the frequently reported influence of the carbon source on biosurfactants production (Abdel‐Mawgoud *et al*., 2011). However, the results of the biosurfactants yield are contradictory to previous studies, which reported glycerol and vegetable oils as superior to petroleum hydrocarbons in promoting biosurfactants production. This contradiction could be due to different culture conditions, different strains and different extraction and purification protocols. It is, nonetheless, worth noting that the biosurfactants yield reported here is a result of non‐optimized culturing conditions. We anticipate that optimization of the culture conditions and recovery protocols may lead to higher yields.

### Rhamnolipids congener profile of the biosurfactants produced from HVGO

HPLC/MS analysis revealed pseudomolecular ions that are consistent with the formation of rhamnolipid biosurfactants (Table 1). This finding is consistent with our previous studies on the AK6U strain (Ismail *et al*., 2014, 2015). A total of 23 different rhamnolipid homologues were produced in detectable levels when *P. aeruginosa* AK6U was grown on HVGO. Thirteen congeners were dirhamnolipids, of which the rhamnosyl–rhamnosyl–β–hydroxydodecanoyl–β–hydroxydodecanoate (Rha–Rha–C_12_–C_12_) and rhamnosyl–rhamnosyl–β–hydroxydecanoyl–β–hydroxydecanoate (Rha–Rha–C_10_–C_10_) congeners were the most abundant (Fig. S3). However, of the two dominant dirhamnolipid homologues, Rha–Rha–C_12_–C_12_ constituted the bulk (61% abundance), while the Rha–Rha–C_10_–C_10_ homologues formed a distant second (10%) of the produced rhamnolipids. Despite the large number of rhamnolipid homologues produced on HVGO, with the exception of Rha–C_8_ (8.2%) and Rha–C_10_–C_12_ (4.9%), most monorhamnolipids were at very low levels (relative abundance values, 0.07–0.38%).

**Table 1:** Relative abundance (%) of rhamnolipid congeners produced from HVGO

	Rhamnolipid congener	Pseudomolecular ion	% in HVGO cultures
1	Rha–C_8_–C_8_	447	0.07
2	Rha–Rha–C_8_–C_8_	593	0.5
3	Rha–Rha–C_8_–C_10_	621	Not detected
4	Rha–Rha–C_10_–C_8_	621	0.29
5	Rha–C_8_–C_10_	475	0.19
6	Rha–C_10_–C_8_	475	1.96
7	Rha–Rha–C_8_–C_12:1_	647	Not detected
8	Rha–Rha–C_12:1_–C_8_	647	3.67
9	Rha–Rha–C_10_–C_10_	649	10
10	Rha–C_10_–C_10_	503	0.77
11	Rha–Rha–C_10_–C_12:1_	675	0.17
12	Rha–C_10_–C_12:1_	529	1.05
13	Rha–C_12:1_–C_10_	529	0.07
14	Rha–Rha–C_12:1_–C_10_	675	1.19
15	Rha–Rha–C_12_–C_10_	677	Not detected
16	Rha–Rha–C_10_–C_12_	677	0.11
17	Rha–C_12_–C_10_	531	4.86
18	Rha–C_10_–C_12_	531	Not detected
19	Rha–Rha–C_12:1_–C_12_	703	0.38
20	Rha–Rha–C_10_–C_14:1_	703	Not detected
21	Rha–Rha–C_12_–C_12_	705	61.11
22	Rha–Rha–C_8_	451	4.45
23	Rha–Rha–C_10_	479	0.07
24	Rha–C_8_	305	8.19
25	Rha–Rha–C_12:1_	505	0.14
26	Rha–Rha–C_12_	507	0.07
27	Rha–C_10_	333	0.32
28	Rha–C_12_	361	0.38
	Total	28	23

The congener profile of the rhamnolipid biosurfactants recovered from the HVGO cultures was different from that reported for other carbon sources. Rhamnolipids produced from different substrates are composed of mixtures of the dirhamnolipid Rha–Rha–C_10_–C_10_ and monorhamnolipid Rha–C_10_–C_10_ (in varying proportions) as the dominant homologues (Henkel *et al*., 2015) To the contrary, the much less frequently reported dirhamnolipid homologues Rha–Rha–C_12_–C_12_ were produced predominantly on HVGO, while Rha–Rha–C_10_–C_10_ congeners constituted a minor fraction. We observed in our earlier investigations that dirhamnolipid congeners dominated biosurfactants produced by AK6U (Ismail *et al*., 2015), which is consistent with our findings in this investigation. However, Ismail *et al*. (2015) found that the most dominant congeners were of the Rha–Rha–C_10_–C_10_ type and no Rha–Rha–C_12_–C_12_ congeners could be detected when AK6U grew on glucose as a carbon source and either dibenzothiophene, dibenzothiophene‐sulfone, or MgSO_4_ as a sulfur source. This is in contrast to the findings in the current study.

The significance of the dominant production of Rha–Rha–C_12_–C_12_ rhamnolipid congeners in the HVGO culture could be envisaged in view of the chemical composition of HVGO and the well‐documented effect of the carbon source on the rhamnolipid congener composition. The long‐chain dodecanoyloxydodecanoate moiety (–C_12_–C_12_) is more hydrophobic in character than the –C_10_–C_10_ moiety and therefore might be more compatible with the characteristics of the hydrophobic long‐chain hydrocarbon components of HVGO (Gray, 1994; Pineda‐Flores and Mesta‐Howard, 2001; Ramirez‐Corredores and Borole, 2006; Pan *et al*., 2015). In addition, the dominance of the –C_12_–C_12_ lipid moiety may be simply a reflection of the unique complex chemical composition of HVGO as a substrate for rhamnolipids production. The production of hydrophobic long‐chain rhamnolipid congeners would enable more effective emulsification to enhance the bioavailability of the complex hydrophobic components present in HVGO. It is known that hydrocarbon solubilization efficiency of surfactants depends on the length and structure of the hydrophobic moiety (tail; Yanto and Tachibana, 2014). In general, the micellar aggregation number and micellar size increase with surfactant tail length. Also, surfactants with isomeric straight chains have better hydrocarbon solubilizing power than those with branched hydrophobic chains, and saturated forms have more solubilizing power than unsaturated forms (Paria and Yuet, 2006). Accordingly, we propose that the dominance of the Rha–Rha–C_12_–C_12_ congeners in the HVGO cultures may represent an adaptation of the AK6U strain to the presence of HVGO as a sole carbon and sulfur source.

As frequently reported in the literature, the type and concentration of the carbon source in the culture medium have a profound effect on the congener composition, yield and activity of the rhamnolipids produced by different bacteria (Arino *et al*., 1996; Mata‐Sandoval *et al*., 2001; Abdel‐Mawgoud *et al*., 2011). Recently, Zhang *et al*. (2014) reported that chain length of fatty acid substrates affects the rhammolipids congener distribution for *P. aeruginosa* ATCC 9027. Moreover, it was also reported that the congener composition influences the activity and efficiency of rhamnolipid biosurfactants. Even minor differences in congener profile can influence solubility, surface activity, emulsification power, biological activity, packing pattern, micelle formation and sensitivity to precipitation from water by counter ions. (Mata‐Sandoval *et al*., 1999; Costa *et al*., 2010; Das *et al*., 2014). Previous work has shown that rhamnolipid mixtures rich in congeners with fatty acyl moiety longer than C_10_ (e.g. Rha–C_10_–C_12_, Rha–C_10_–C_12:1_) had a lower CMC and caused stronger reduction in surface tension than those rich in congeners with shorter chains (Mata‐Sandoval *et al*., 1999; Costa *et al*., 2010).

### Changes in the chemical composition of HVGO

The second main objective of our study was to explore the HVGO biocatalytic upgrading potential of the *P. aeruginosa* AK6U strain via investigating whether bacterial growth can bring about changes in the chemical composition of HVGO. After incubation of the AK6U strain with HVGO in MSM as a carbon and sulfur source, HVGO was recovered from the cultures, and the maltene fraction was separated and subjected to SimDist analysis to explore any changes in the major distillation fractions. Moreover, detailed analysis on the separated maltene fractions was conducted by TOF‐MS and FT‐ICR‐MS to unravel changes in the chemical composition.

The SimDist analysis of the biotreated HVGO revealed a relative decrease in the high‐boiling point (BP) heavy fuel oil fraction (BP 426–565 °C) and increase in the lighter distillate diesel fraction (BP 315–426 °C), compared with the abiotic control HVGO (Fig. S4). Nonetheless, the differences were statistically insignificant (*p *>* *0.05). Approximately, the same amounts of the remaining highly viscous residue (BP > 565 °C) and the lighter kerosene (BP 204–315 °C) were obtained in both of the abiotic control and biotreated HVGO (Fig. S4). In a recent study on crude oil, Noh *et al*. (2012) reported that incubation of crude oil with a culture of rhamnolipids‐producing *P. aeruginosa* improved the oil distillation process in terms of shorter distillation time, lower temperature range and higher distillate volume. The authors attributed the enhanced distillation efficiency to hydrocarbon emulsification power of the rhamnolipid biosurfactants and hydrocarbon degradation by *P. aeruginosa*.

**Figure 3:**
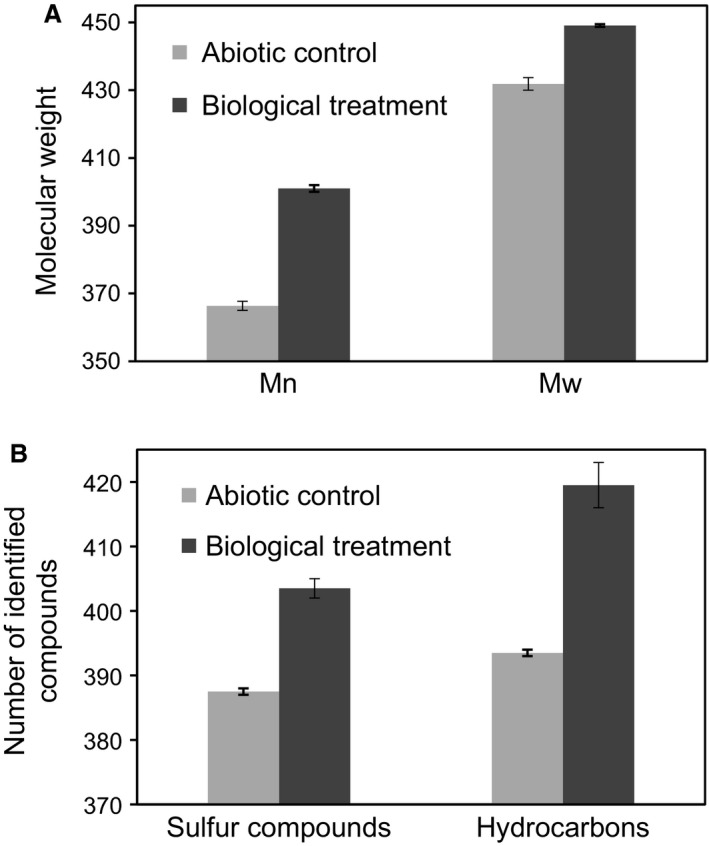
A. The calculated number‐average (Mn) and weight‐average (Mw) molecular weights of the chemical species detected in the maltene fractions extracted from abiotic control and biotreated HVGO. B. Absolute number of sulfur‐containing and hydrocarbon compounds identified in the maltene fractions extracted from abiotic control and biotreated HVGO.

The TOF‐MS spectra of the maltene fraction recovered from the abiotic control and biotreated HVGO exhibited nearly identical molecular weight distributions of hydrocarbons with hydrocarbon signals mainly between *m/z* > 140 and *m/z* < 800 (data not shown). However, the plot of normalized cumulative MS abundance versus the *m/z* ratio showed a relative decrease in lower molecular weight species (*m/z* below 350) in the biotreated maltene fraction. The Mn and Mw values increased significantly (*p *<* *0.05) in the biotreated maltene fraction (Fig. 3A).

The FT‐ICR‐MS analysis was conducted to get more information on the different aromatic species in the maltene fractions. The main heteroatom and aromatic classes included hydrocarbons, sulfur‐, disulfur‐, nitrogen‐ and oxygen‐containing species, with sulfur compounds and hydrocarbons comprising approximately 70% of the aromatic species. The absolute number of the identified sulfur compounds and hydrocarbon species was significantly higher (*p *<* *0.05) in the biotreated maltene fraction compared with the abiotic control (Fig. 3B). The increase in Mn and Mw suggests that some of the asphaltene species (i.e. those with high molecular weight) were mobilized into the maltene fraction of the biotreated HVGO. This could be attributed to physical solubilization of these components by biosurfactants produced by the AK6U strain and/or the biodegradation/biotransformation of high molecular weight pentane‐insoluble asphaltene species (such as the polycyclic aromatics with disulfide bonds) into slightly lighter pentane‐soluble species in the maltene fraction. Biosurfactants are efficient solubilizing or mobilizing agents for different hydrocarbons (Ward, 2010). Moreover, various *Pseudomonas* spp., including *P. aeruginosa*, have been implicated in the biodegradation of crude oil, aromatic and aliphatic hydrocarbons as well as biosurfactants production (Mnif *et al*., 2011; Liu *et al*., 2012; Banat *et al*., 2014; Ismail *et al*., 2015; Chettri *et al*., 2016; Jin *et al*., 2016). In a recent study on asphaltenes biodegradation by *P. aeruginosa* and other bacteria, Ali *et al*. (2012) reported the biotransformation of the organosulfur moieties in asphaltene to sulfones and sulfoxides. These results are in line with our suggestion that the *P. aeruginosa* AK6U strain utilized HVGO as a sulfur source, which might be a significant biomodification step towards the depolymerization/biodegradation of asphaltenes (Ali *et al*., 2012). Nonetheless, we cannot exclude the possibility that the compositional changes in the maltene fraction are due to biocatalysis on the maltene components.

Other major findings of our studies are the increase in the number of hydrocarbon and sulfur compounds in the biotreated maltene fraction as per the FT‐ICR‐MS analysis, as well as the relative decrease in the amount of heavy fuel oil (high molecular weight, high BP) as per the SimDist analysis. These results provide further evidence for the capability of the AK6U strain to transform and upgrade different high molecular weight hydrocarbon classes including the sulfur‐containing compounds in HVGO into lighter components. Jahromi *et al*. (2014) reported up to 51.5% biodegradation of asphaltene fraction after 2 months by different bacteria including *P. fluorescens* and *P. aeruginosa*. In a similar context, Ali *et al*. (2012) reported the biodegradation of asphaltenic fraction (extracted from crude oil) by different halotolerant bacteria including a *P. aeruginosa* strain. FT‐IR and gel permeation chromatography revealed alterations in functional groups and reduction in average molecular weight of the biologically treated asphaltenes.

**Figure 4:**
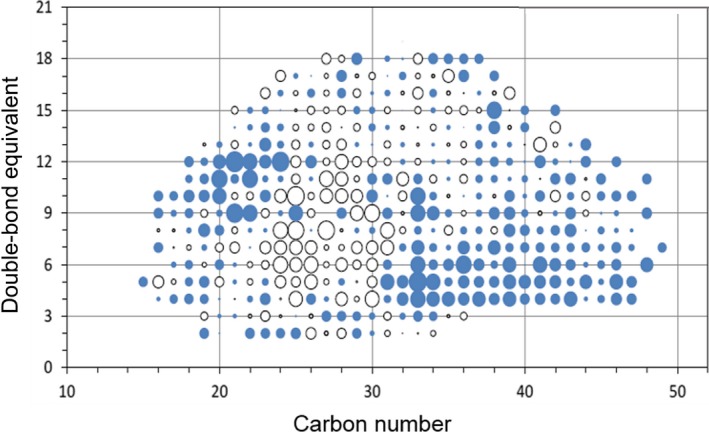
Difference map of hydrocarbon species. Blue dots represent species relatively enriched in the maltene fraction of the biotreated HVGO, and white dots represent species that are relatively more abundant in the maltene fraction of the abiotic control HVGO. The size of the dot reflects the abundance of a compound in the respective maltene fraction.

The elucidation of the exact chemical composition of the hydrocarbon species, which were probably mobilized from the asphaltene fraction to the maltene fraction in the biotreated HVGO is very challenging due to the complexity of analysing the asphaltene fraction. The results of the FT‐ICR‐MS confirmed the TOF‐MS data indicating that more sulfur and hydrocarbon species were enriched in the maltene fraction of the biotreated HVGO. Interestingly, a difference map constructed by plotting the double‐bond equivalent (DBE) versus the number of carbon atoms (C) of the aromatic species revealed obvious differences in the distribution of these compounds in the abiotic control and biotreated HVGO (Fig. 4). Two regions delimiting the abundance and distribution of most of the aromatic species in the maltene fraction of the biotreated HVGO were recognized, a region abundant in high C‐number aromatics (C > 32) with low DBE (DBE ˂ 7) and a region abundant in low C‐number aromatics (C ≤ 22) with high DBE (DBE ≥ 9). In contrast, most of the aromatic species in the abiotic control maltene fraction were concentrated nearly in the middle of the map in one area defining aromatics with C ≤ 30 and DBE in the range 4–12. All the analytical results are coherent and together confirm that the biocatalytic machinery of the AK6U strain worked under ambient conditions as a biorefinery via biocatalytic upgrading of HVGO. The fractional distillation of the maltene fractions using SimDist revealed a relative decrease in the heavy fuel oil concomitant with increase in the lighter diesel fraction. Although this pattern makes sense, the differences between the biotreated and the abiotic control HVGO were statistically insignificant. It appears that the HVGO compositional changes mediated by the AK6U strain were not sufficient to induce significant change in the distillation profile of the biotreated HVGO. However, on the industrial refining scale where huge volumes of the oil are processed, even small differences might lead to considerably increased yield, which further improves the revenue capture of the refining process. The results point to the need for deeper understanding of the bioupgrading mechanisms and comprehensive analyses of HVGO to enable the design of more efficient bioprocess.

Studies on the bioconversion or biocatalytic upgrading of HVGO are largely lacking in the literature. As discussed throughout this manuscript, the majority of the studies focused on the biodegradation/biotransformation of asphaltene fractions extracted from crude oil. The interest in asphaltene is based on the fact that it is the heaviest, most complex and most biodegradation‐resistant fraction of crude oil. In addition to the literature mentioned earlier, recently Pan *et al*. (2015) applied pyrolysis gas chromatography to study the composition of asphaltene fractions precipitated from biodegraded bitumens. The results revealed biodegradation of linear alkyl moieties, *n*‐fatty acids and aliphatic alcohols that are bound to the asphaltene core. Although some of the previous studies showed the susceptibility of asphaltenes to microbial attack, it is also important to study real hydrocarbon streams like HVGO, VR, in addition to heavy crude oil, to enable the development of various technologies such as biocatalytic upgrading/bioconversion, microbial enhanced oil recovery and bioremediation. In a recent study, Maass *et al*. (2015) reported the desulfurization and denitrogenation of HVGO by *Rhodococcus erythropolis* ATCC 4277. This is also in agreement with the observed ability of the *P. aeruginosa* AK6U strain to utilize HVGO as a sulfur source. In addition, there are some studies on crude oil. FT‐IR analysis revealed the enzymatic oxidation of carbon and sulfur atoms in both maltenes and asphaltenes from extraheavy crude oil treated with a cell‐free laccase produced by the fungus *Pestalotiopsis palmarum* BM‐04 (Naranjo‐Briceño *et al*., 2013).

The data presented here raise different questions that represent the directions for future investigations. For instance, it is important to optimize the biosurfactants production conditions and understand the involved biosynthetic machinery when HVGO is provided as a carbon source. We are currently performing comparative qPCR studies to investigate the expression of the biosurfactants production and regulation (quorum‐sensing) genes using HVGO as a carbon source. Furthermore, it is also important to conduct economic viability studies to explore the relevance of HVGO as a feedstock for industrial application. Future research should also focus on the catabolic pathways involved in the biotransformation/biodegradation of different HVGO components as well as the underlying genes and enzymes. This should include in‐depth structural investigations on the different HVGO components in addition to the application of systems biology studies such as proteomics and metabolomics. As per the structural complexity and heterogeneity of HVGO, the application of mixed microbial cultures would be also interesting. The role of biosurfactants in the upgrading process is also worth investigating. Optimization of the bioupgrading conditions is essential to explore the potential of promoting the HVGO‐derived distillates yield significantly. Eventually, it is also interesting to investigate the bioupgrading potential of the vacuum residue (VR), whose physicochemical properties are even more challenging than HVGO.

## Conclusions

We presented data supporting the potential of *P. aeruginosa* AK6U as a biocatalyst for valorization of HVGO. The first aspect underlies the bioconversion of HVGO to value‐added products such as the high‐value rhamnolipid biosurfactants. The rhamnolipid congeners’ profile was dominated by the less frequently reported dirhamnolipids Rha–Rha–C_12_–C_12_, which might be a reflection of the HVGO chemical composition. The second valorization aspect is based on bioupgrading of HVGO resulting in compositional changes that were facilitated or mediated by the biocatalytic machinery of the AK6U strain.

## Experimental procedures

### HVGO and the bacterial strain

*Pseudomonas aeruginosa* AK6U was used for HVGO bioconversion and bioupgrading experiments. This strain was recently isolated from hydrocarbons‐polluted soil and characterised in our laboratory. It produces rhamnolipid biosurfactants from various carbon sources. Moreover, biosurfactants production is enhanced when the AK6U strain is provided with thiophenic organosulfur compounds instead of inorganic sulfate as a sulfur source (AlKhalifa, 2013; Ismail *et al*., 2014, 2015). HVGO was provided by Bahrain Petroleum Company (BAPCO, Bahrain).

### Culture media and growth conditions

Commercially available Luria‐Bertani (LB) agar and broth media were prepared according to manufacturer's instructions. Sulfur‐free mineral salts medium (MSM) had the following composition (per litter of deionized water): KH_2_PO_4_, 1.08 g; K_2_HPO_4_, 5.6 g; NH_4_Cl, 0.54 g; MgCl_2_.6H_2_O, 0.2 g; CaCl_2_.2H_2_O, 0.044 g; FeCl_2_.4H_2_O, 1.5 mg; vitamins (cyanocobalamin 0.2 mg, pyridoxine‐HCl 0.6 mg, thiamine‐HCl 0.4 mg, nicotinic acid 0.4 mg, *p*‐aminobenzoate 0.32 mg, biotin 0.04 mg and Ca‐pantothenate 0.4 mg) and trace elements (ZnCl_2_.7H_2_O 70 μg, MnCl_2_.4H_2_O 100 μg, CuCl_2_ 20 μg, CoCl_2_.6H_2_O 200 μg, Na_2_MoO_4_.2H_2_O 40 μg, NiCl_2_.6H_2_O 20 μg and H_3_BO_3_ 20 μg). HVGO was autoclaved and added to MSM as both carbon and sulfur source (20% v/v). All liquid cultures were incubated in an orbital shaker (200 rpm) at 30 °C. All cultures on solid media were incubated at 30 °C for 48 h. Liquid cultures were routinely grown in 250‐ml Erlenmeyer flasks containing 100 ml of the growth medium. Uninoculated medium was included as a negative control to compensate for abiotic losses. To measure bacterial growth at time intervals, the cultures were brought out of the incubator and kept for a few minutes to allow separation of the emulsions (whenever possible). Then, culture samples (30 ml) were retrieved from the lower (aqueous) phase, and the cells were harvested by centrifugation at 10 000 rpm for 5 min. The cell pellets were then dried to constant weight in a drying oven at 105 °C.

### Preparation of starter cultures

The AK6U strain was inoculated into 1‐l Erlenmeyer flasks (in duplicates) containing autoclaved 400 ml of LB‐broth and incubated for 16 h. The bacterial cells were harvested by centrifugation in 500‐ml plastic centrifuge tubes (10 000 rpm, 10 min). The cell pellets were then washed once (on ice) with 40 ml of ice‐cold 0.1 M potassium phosphate buffer (pH 7) and resuspended in 10 ml of the same buffer, and the two cell suspensions were pooled. This cell suspension was used as the inoculum for the bioupgrading and bioconversion experiments.

### HVGO bioconversion and bioupgrading experiments

Experiments for bioupgrading and bioconversion of HVGO to biosurfactants were performed in MSM cultures containing 20% (v/v) of autoclaved HVGO as a sole carbon and sulfur source. The culture medium (800 ml) and HVGO (200 ml) were dispensed into 2‐l Erlenmeyer flasks (duplicates), followed by inoculation with 9 ml of the cell suspension to reach biomass density of o.35 g dry cell weigh (dcw) l^−1^. To monitor abiotic losses of HVGO, uninoculated flasks containing MSM and HVGO were also included. All cultures were incubated for 56 days at 200 rpm and 30 °C. Cultures were inspected visually for turbidity, oil emulsification/dispersion and foaming. Growth was followed by measuring the biomass dry weight at time intervals. Biosurfactants production was followed by measuring the temporal changes in surface tension of cell‐free culture supernatants as described (Ismail *et al*., 2014).

### Recovery of the crude biosurfactants from HVGO cultures

Cell‐free culture supernatants (50 ml) were collected by centrifugation (14 000 rpm, 10 min) after 18 and 39 days. After acidification to pH 2 with 25% HCl, the supernatants were kept at 4 °C overnight. The biosurfactants were extracted twice with one volume of chloroform–methanol (2:1) in a separating funnel. The organic phases were pooled and evaporated under vacuum (Buchi rotary evaporator V850, Switzerland) at 40 °C. The residue was weighed, and the crude biosurfactants yield was estimated as g l^−1^. The analysis of the rhamnolipids congener profile was performed on cell‐free culture supernatants collected after 39 days of incubation as described (Ismail *et al*., 2015).

### Analysis of the chemical composition of HVGO

Heavy vacuum gas oil was recovered from the AK6U cultures and the abiotic controls after 56 days of incubation. The whole contents of the cultures were centrifuged for 10 min at 10 000 rpm to separate the oil from the aqueous phase. Then, the oil was transferred to clean bottles and subjected to the following analyses.

### Separation of the maltene and asphaltene fractions of HVGO

Samples of biotreated HVGO as well as of control HVGO were subjected to solvent extraction process to separate the asphaltene (n‐pentane‐insoluble) and the maltene (n‐pentane‐soluble) fractions as follows: In a 2‐l flask containing 10 ml of HVGO, 400 ml of n‐pentane was added. The flask was sealed with a rubber stopper wrapped with aluminium foil, to avoid direct contact of stopper with the solvent, and the mixture was shaken vigorously. Then, the mixture was kept to equilibrate for 2 days at room temperature with occasional shaking 3–4 times during this period. After ageing for 2 days, the mixture was filtered carefully through a preweighed 0.22 μm filter (MF‐Millipore, mixed cellulose ester membrane filter) using vacuum in a preweighed filtration flask. Both of the precipitated asphaltene fraction on the membrane filter and the pentane‐soluble maltene fraction in the filtration flask were subjected to air drying under hood. After complete drying, the weights of the asphaltene and the maltene fractions were measured. For further analyses, the asphaltene fraction precipitated on the membrane filter was recovered by washing and vigorous shaking with toluene, and the dried maltene fraction in the filtration flask was also dissolved in toluene at a concentration of 1 g/100 ml toluene.

### Simulated distillation (SimDist)

Determination of the boiling point (BP) distribution of the distillate fractions using SimDist is essential for monitoring and controlling refining processes as well as for product specification testing of petroleum fractions such as HVGO. The maltene fractions of the control and the biotreated HVGO were subjected to SimDist analysis following the American Society for Testing and Materials method (ASTM D‐7169, Austrich *et al*., 2015). The high temperature SimDist system based on the 6890N GC (Agilent Technologies, Santa Clara, California USA) for heavy oil fractions was used. This fractional distillation analysis can monitor shifts in the different fuel fractions due to changes in the composition of the different chemical classes.

### Fourier transform ion cyclotron resonance mass spectrometry (FT‐ICR MS)

Comprehensive analysis on the aromatic and heteroatom compounds distributions in the maltene fraction of HVGO was conducted using 15T SolariX FT‐ICR mass spectrometer as previously described (Gómez‐Escudero *et al*., 2015). Stock sample solutions were prepared in toluene at a concentration of 10 mg ml^−1^.

### Time‐of‐flight mass spectrometry (TOF‐MS)

We conducted TOF‐MS analysis to calculate the number‐average (Mn) and weight‐average (Mw) molecular weights of the biotreated maltene fraction. TOF‐MS analysis was conducted using the Xevo G2‐XS Tof Time‐of‐Flight Mass Spectrometry (Waters, USA), which combines StepWave ion optics, XS Collision Cell and QuanTof technology. Ions are accelerated by an electric field, and the time that different ions take to reach the detector at a known distance, which is dependent on the mass‐to‐charge ratio of the ions, is measured. Data were recorded for one minute, and the mass spectra of the distributed ions were obtained.

### Statistical analysis

One way analysis of variance (Tukey test) was performed with the JMP statistical software (version 13; SAS Corporation, Chicago, IL, USA). Statistical significance was defined as *p *<* *0.05.

## Conflict of interest

The authors have no conflict of interest to declare.

## Supporting information


**Fig. S1.** Temporal changes in color, turbidity, and oil consistency in *P. aeruginosa* AK6U cultures containing 20% HVGO (v/v) as the sole carbon and sulfur source.

**Fig. S2.** Centrifuge bottles showing the cultures of *P. aeruginosa* AK6U on HVGO (56 days of incubation) after centrifugation at 10 000 rpm for 10 min.

**Fig. S3.** A mass spectrum of a rhamnolipid produced by *P. aeruginosa* AK6U grown on HVGO (20% v/v) in mineral salts medium as the sole carbon and sulfur source.

**Fig. S4.** Results of fractional distillation (SimDist) analysis of maltene fractions, extracted from abiotic control and bio‐treated HVGO, into different boiling point fractions (recovered weight %). [Residue = bottom of the barrel fraction with very high BP (> 565 °C), Fuel = heavy viscous fuel oil (BP ≈ 426–565 °C), Diesel fraction (BP ≈ 315–426 °C), Kerosene fraction (BP ≈ 204–315 °C)]

## References

[mbt212741-bib-0001] Abdel‐Mawgoud, A.M. , Hausmann, R. , Lepine, F. , Muller, M.M. , and Deziel, E. (2011) Rhamnolipids: detection, analysis, biosynthesis, genetic regulation, and bioengineering of production In Biosurfactants: From Genes to Applications. Soberon‐Chavez G. (ed.). Berlin: Springer‐Verlag, pp. 13–55.

[mbt212741-bib-0002] Ali, H.R. , El‐Gendy, N. , Moustafa, Y.M. , Roushdy, M.I. and Hashem, A.I. (2012) Degradation of asphaltenic fraction by locally isolated halotolerant bacterial strains. *ISRN Soil Science* 2012.

[mbt212741-bib-0003] AlKhalifa, F.S. (2013) Effect of the carbon sources on biosurfactants production by bacteria isolated from industrial wastewater and soil polluted with lubricating oil. MSc thesis. Arabian Gulf University, Bahrain.

[mbt212741-bib-0004] Arino, S. , Marchal, R. , and Vandecasteele, J.‐P. (1996) Identification and production of a rhamnolipidic biosurfactant by a *Pseudomonas* species. Appl Microbiol Biotechnol 45: 162–168.

[mbt212741-bib-0005] Austrich, A.J. , Buenrostro‐Gonzalez, E. , and Lira‐Galeana, C. (2015) ASTM D‐5307 and ASTM D‐7169 SIMDIS standards: a comparison and correlation of methods. Pet Sci Tech 33: 657–663.

[mbt212741-bib-0006] Ayala, M. , Hernández‐López, E.L. , Perezgasga, L. , and Vazquez‐Duhalt, R. (2012) Reduced coke formation and aromaticity due to chloroperoxidase‐catalyzed transformation of asphaltenes from Maya crude oil. Fuel 92: 245–249.

[mbt212741-bib-0007] Banat, I.M. , Satpute, S.K. , Cameotra, S.S. , Patil, R. , and Nyayanit, N.V. (2014) Cost effective technologies and renewable substrates for biosurfactants production. Front Microbiol 5: 697. 25566213 10.3389/fmicb.2014.00697 PMC4264478

[mbt212741-bib-0008] Bredholt, H. , Josefsen, K. , Vatland, A. , Bruheim, P. , and Eimhjellen, K. (1998) Emulsification of crude oil by an alkane‐oxidizing *Rhodococcus* species isolated from seawater. Can J Microbiol 44: 330–340.

[mbt212741-bib-0009] Chettri, B. , Mukherjee, A. , Langpoklakpam, J.S. , Chattopadhyay, D. , and Singh, A.K. (2016) Kinetics of nutrient enhanced crude oil degradation by *Pseudomonas aeruginosa* AKS1 and *Bacillus* sp. AKS2 isolated from Guwahati refinery, India. Environ Pollut 216: 548–558. 27317496 10.1016/j.envpol.2016.06.008

[mbt212741-bib-0010] Costa, S.G. , Nitschke, M. , Lepine, F. , Deziel, E. , and Contiero, J. (2010) Structure, properties and applications of rhamnolipids produced by *Pseudomonas aeruginosa* L2‐1 from cassava wastewater. Process Biochem 45: 1511–1516.

[mbt212741-bib-0011] Das, P. , Yang, X.‐P. , and Ma, L.Z. (2014) Analysis of biosurfactants from industrially viable *Pseudomonas* strain isolated from crude oil suggests how rhamnolipids congeners affect emulsification property and antimicrobial activity. Front Microbiol 5: 696. 25566212 10.3389/fmicb.2014.00696 PMC4273660

[mbt212741-bib-0012] De Almeida, D.G. , Soares Da Silva, R.C. , Luna, J.M. , Rufino, R.D. , Santos, V.A. , Banat, I.M. , and Sarubbo, L.A. (2016) Biosurfactants: promising molecules for petroleum biotechnology advances. Front Microbiol 7: 1718. 27843439 10.3389/fmicb.2016.01718 PMC5087163

[mbt212741-bib-0013] Fedorak, P.M. , Semple, K.M. , Vazquez‐Duhalt, R. , and Wastlake, D.W.S. (1993) Chloroperoxidase mediated modifications of petroporphyrins and asphaltenes. Enzyme Microb Technol 15: 429–437. 10.1016/0141-0229(93)90169-3 7764253

[mbt212741-bib-0014] Feng, W. , Swift, S. , and Singhal, N. (2013) Effects of surfactants on cell surface tension parameters and hydrophobicity of *Pseudomonas putida* 852 and *Rhodococcus erythropolis* 3586. Colloids Surf B Biointerfaces 105: 43–50. 23352946 10.1016/j.colsurfb.2012.12.034

[mbt212741-bib-0015] Garcia‐Arellano, H. , Buenrostro‐Gonzalez, E. , and Vazquez‐Duhalt, R. (2004) Biocatalytic transformation of petroporphyrins by chemical modified cytochrome C. Biotechnol Bioeng 85: 790–798. 14991657 10.1002/bit.20023

[mbt212741-bib-0016] Gómez‐Escudero, A. , Rojas‐Ruíz, F.A. , and Orrego‐Ruíz, J.A. (2015) Characterization of vacuum gas oils using FT‐ICR MS. CT&F‐ Ciencia Tecnol Futur 6: 69–80.

[mbt212741-bib-0017] Gray, M.R. (1994) Heavy oil and residue properties and composition In Upgrading Petroleum Residue and Heavy Oils. New York: Marcel Dekker Inc., pp. 1–40.

[mbt212741-bib-0018] Henkel, M. , Syldatk, C. , and Hausmann, R. (2015) The prospects for the production of rhamnolipids on renewable resources‐evaluation of novel feedstocks and perspectives of strain engineering In Biosurfactants Production and Utilization‐Processes, Technologies, and Economics. Kosaric N., and Vardar‐Sukan F. (eds). New York: CRC Press, pp. 83–99.

[mbt212741-bib-0019] Hernández‐López, E.L. , Ramírez‐Puebla, S.T. , and Vazquez‐Duhalt, R. (2015) Microarray analysis of *Neosartorya fischeri* using different carbon sources, petroleum asphaltenes and glucose‐peptone. Genom Data 5: 235–237. 26484261 10.1016/j.gdata.2015.06.013 PMC4583671

[mbt212741-bib-0020] Ismail, W. , El Nayal, A. , Ramadan, A. and Abotalib, N. (2014) Sulfur source‐mediated transcriptional regulation of the *rhlABC* genes involved in biosurfactants production by *Pseudomonas* sp. strain AK6U. Front Microbiol 5, 423. 25177318 10.3389/fmicb.2014.00423 PMC4132291

[mbt212741-bib-0021] Ismail, W. , Al Shammary, S. , El‐Sayed, W.S. , Obuekwe, C. , El Nayal, A.M. , Abdul Raheem, A.S. and Al‐Humam, A. (2015) Stimulation of rhamnolipid biosurfactants production in *Pseudomonas aeruginosa* AK6U by organosulfur compounds provided as sulfur sources. Biotechnol Rep 7, 55–63. 10.1016/j.btre.2015.03.001 PMC5466058 28626715

[mbt212741-bib-0022] Jahromi, H. , Fazaelipoor, M.H. , Ayatollahi, S. , and Niazi, A. (2014) Asphaltenes biodegradation under shaking and static conditions. Fuel 117: 230–235.

[mbt212741-bib-0023] Jin, J. , Yao, J. , and Zhang, Q. (2016) Biodegradation of phenanthrene by *Pseudomonas* sp. JPN2 and structure‐based degrading mechanism study. Bull Environ Contam Toxicol 97: 689–694. 27631505 10.1007/s00128-016-1917-1

[mbt212741-bib-0024] Kilbane, J.J. II (2006) Microbial biocatalyst developments to upgrade fossil fuels. Curr Opin Biotech 17: 305–314. 16678400 10.1016/j.copbio.2006.04.005

[mbt212741-bib-0025] Kumar, C.G. , Mamidyala, S.K. , Sujitha, P. , Muluka, H. , and Akkenapally, S. (2012) Evaluation of critical nutritional parameters and their significance in the production of rhamnolipid biosurfactants from *Pseudomonas aeruginosa* BS‐161R. Biotechnol Prog 28: 1507–1516. 22961871 10.1002/btpr.1634

[mbt212741-bib-0026] Le Borgne, S. , and Quintero, E. (2003) Biotechnological processes for the refining of petroleum. Fuel Process Technol 81: 155–169.

[mbt212741-bib-0027] Liu, H. , Liang, R. , Tao, F. , Ma, C. , Liu, Y. , Liu, X. , and Liu, J. (2012) Genome sequence of *Pseudomonas aeruginosa* strain SJTD‐1, a bacterium capable of degrading long‐chain alkanes and crude oil. J Bacteriol 194: 4783–4784. 22887679 10.1128/JB.01061-12 PMC3415508

[mbt212741-bib-0028] Maass, D. , Todescato, D. , Moritz, D.E. , Oliveira, J.V. , Oliveira, D. , Ulson de Souza, A.A. , *et al* (2015) Desulfurization and denitrogenation of heavy gas oil by *Rhodococcus erythropolis* ATCC 4277. Bioprocess Biosyst Eng 38: 1447–1453. 25759162 10.1007/s00449-015-1386-7

[mbt212741-bib-0029] Mata‐Sandoval, J.C. , Karns, J. , and Torrents, A. (1999) High‐performance liquid chromatography method for the characterization of rhamnolipid mixtures produced by *Pseudomonas aeruginosa* UG2 on corn oil. J Chromatogr A 864: 211–220. 10669288 10.1016/s0021-9673(99)00979-6

[mbt212741-bib-0030] Mata‐Sandoval, J.C. , Karns, J. , and Torrents, A. (2001) Effect of nutritional and environmental conditions on the production and composition of rhamnolipids by *P. aeruginosa* UG2. Microbiol Res 155: 249–256. 11297354 10.1016/S0944-5013(01)80001-X

[mbt212741-bib-0031] Mazaheri, A.M. , and Tabatabaee, M.S. (2010) Biosurfactants and their use in upgrading petroleum vacuum distillation residue. Int J Environ Res 4: 549–572.

[mbt212741-bib-0032] Mnif, S. , Chamkha, M. , Labat, M. , and Sayadi, S. (2011) Simultaneous hydrocarbon biodegradation and biosurfactant production by oilfield‐selected bacteria. J Appl Microbiol 111: 525–536. 21668593 10.1111/j.1365-2672.2011.05071.x

[mbt212741-bib-0033] Naranjo, L. , Urbina, H. , De Sisto, A. , and Leon, V. (2007) Isolation of autochthonous non‐white rot fungi with potential for enzymatic upgrading of Venezuelan extra‐heavy crude oil. Biocatal Biotransformation 25: 341–349. 18833334 10.1080/10242420701379908 PMC2556186

[mbt212741-bib-0034] Naranjo‐Briceño, L. , Pernía, B. , Guerra, M. , Demey, J.R. , De Sisto, A. , Inojosa, Y. , *et al* (2013) Potential role of oxidative exoenzymes of the extremophilic fungus *Pestalotiopsis palmarum* BM‐04 in biotransformation of extra‐heavy crude oil. Microb Biotechnol 6: 720–730. 23815379 10.1111/1751-7915.12067 PMC3815938

[mbt212741-bib-0035] Noh, N.A. , Abdullah, A.A. , Ibrahim, M.N. , and Yahya, A.R. (2012) Rhamnolipid produced by *Pseudomonas aeruginosa* USM‐AR2 facilitates crude oil distillation. J Gen Appl Microbiol 58: 153–161. 22688247 10.2323/jgam.58.153

[mbt212741-bib-0036] Obuekwe, C.O. , Al‐Jadi, Z.K. , and Al‐Saleh, E.S. (2009) Hydrocarbon degradation in relation to cell‐surface hydrophobicity among bacterial hydrocarbon degraders from petroleum‐contaminated Kuwait desert environment. Int Biodeterior Biodegradation 63: 273–279.

[mbt212741-bib-0037] Pan, Y. , Liao, Y. , and Zheng, Y. (2015) Effect of biodegradation on the molecular composition and structure of asphaltenes: clues from quantitative Py–GC and THM–GC. Org Geochem 86: 32–44.

[mbt212741-bib-0038] Paria, S. , and Yuet, P.K. (2006) Solubilization of naphthalene by pure and mixed surfactants. Ind Eng Chem Res 45: 3552–3558.

[mbt212741-bib-0039] Pineda‐Flores, G. , and Mesta‐Howard, A.M. (2001) Petroleum asphaltenes: generated problematic and possible biodegradation mechanisms. Rev Latinoam Microbiol 43: 143–150. 17061501

[mbt212741-bib-0040] Pineda‐Flores, G. , Boll‐Argüello, G. , Lira‐Galeana, C. , and Mesta‐Howard, A.M. (2004) A microbial consortium isolated from a crude oil sample that uses asphaltenes as a carbon and energy source. Biodegradation 15: 145–151. 15228072 10.1023/b:biod.0000026476.03744.bb

[mbt212741-bib-0041] Rahman, K. , Rahman, T. , and McClean, S. (2002) Rhamnolipid biosurfactant production by strains of *Pseudomonas aeruginosa* using low‐cost raw materials. Biotechnol Prog 18: 1277–1281. 12467462 10.1021/bp020071x

[mbt212741-bib-0042] Ramirez‐Corredores, M.M. , and Borole, A.P. (2006) Conventional refining processes In Biocatalysis in Oil Refining. Advisory editors (Delmon B. and Yates J. T.), Series Editor (Centi G.). Vol 164. New York: Elsevier, pp. 9–64.

[mbt212741-bib-0043] Sahu, R. , Song, B.J. , Im, J.S. , Jeon, Y.‐P. , and Lee, C.W. (2015) A review of recent advances in catalytic hydrocracking of heavy residues. J Ind Eng Chem 27: 12–24.

[mbt212741-bib-0044] Soberón‐Chávez, G. , and Maier, R.M. (2011) Biosurfactants: a general overview In Biosurfactants: From Genes to Applications. Soberón‐Chávez G. (ed.). Berlin: Springer‐Verlag, 164: pp. 1–11.

[mbt212741-bib-0045] Speight, J.G. (2013) Refining heavy oil and extra‐heavy oil In Heavy and Extra‐Heavy Oil Upgrading Technologies. New York: Elsevier, pp. 1–14.

[mbt212741-bib-0046] Uribe‐Alvarez, C. , Ayala, M. , Perezgasga, L. , Naranjo, L. , Urbina, H. , and Vazquez‐Duhalt, R. (2011) First evidence of mineralization of petroleum asphaltenes by a strain of *Neosartorya fischeri* . Microb Biotechnol 4: 663–672. 21624102 10.1111/j.1751-7915.2011.00269.x PMC3819015

[mbt212741-bib-0047] Ward, O.P. (2010) Microbial biosurfactants and biodegradation. Adv Exp Med Biol 672: 65–74. 20545274 10.1007/978-1-4419-5979-9_5

[mbt212741-bib-0048] Warne, Z.C. , Ghoshal, S. , and Tufenkji, N. (2010) Bacterial adhesion to hydrocarbons: role of asphaltenes and resins. Colloids Surf B Biointerfaces 79: 219–226. 20452190 10.1016/j.colsurfb.2010.03.054

[mbt212741-bib-0049] Yanto, D.H.Y. , and Tachibana, S. (2014) Enhanced biodegradation of asphalt in the presence of tween surfactants, Mn^2+^ and H_2_O_2_ by *Pestalotiopsis* sp. in liquid medium and soil. Chemosphere 103: 105–113. 24331036 10.1016/j.chemosphere.2013.11.044

[mbt212741-bib-0050] Zhang, L. , Pemberton, J.E. , and Maier, R.M. (2014) Effect of fatty acid substrate chain length on *Pseudomonas aeruginosa* ATCC 9027 monorhamnolipid yield and congener distribution. Process Biochem 49: 989–995.

