# Supplementary material for: Simultaneous valorization and biocatalytic upgrading of heavy vacuum gas oil by the biosurfactant‐producing Pseudomonas aeruginosa AK6U
Source: Microb Biotechnol. 2017 Jul 11;10(6):1628–39. doi: 10.1111/1751-7915.12741 (PMC5658591; doi:10.1111/1751-7915.12741)
Supplement: Supplementary file 1 — Fig. S1. Temporal changes in color, turbidity, and oil consistency in P. aeruginosa AK6U cultures containing 20% HVGO (v/v) as the sole carbon and sulfur source. [file MBT2-10-1628-s001.ppt]

## Slide 1
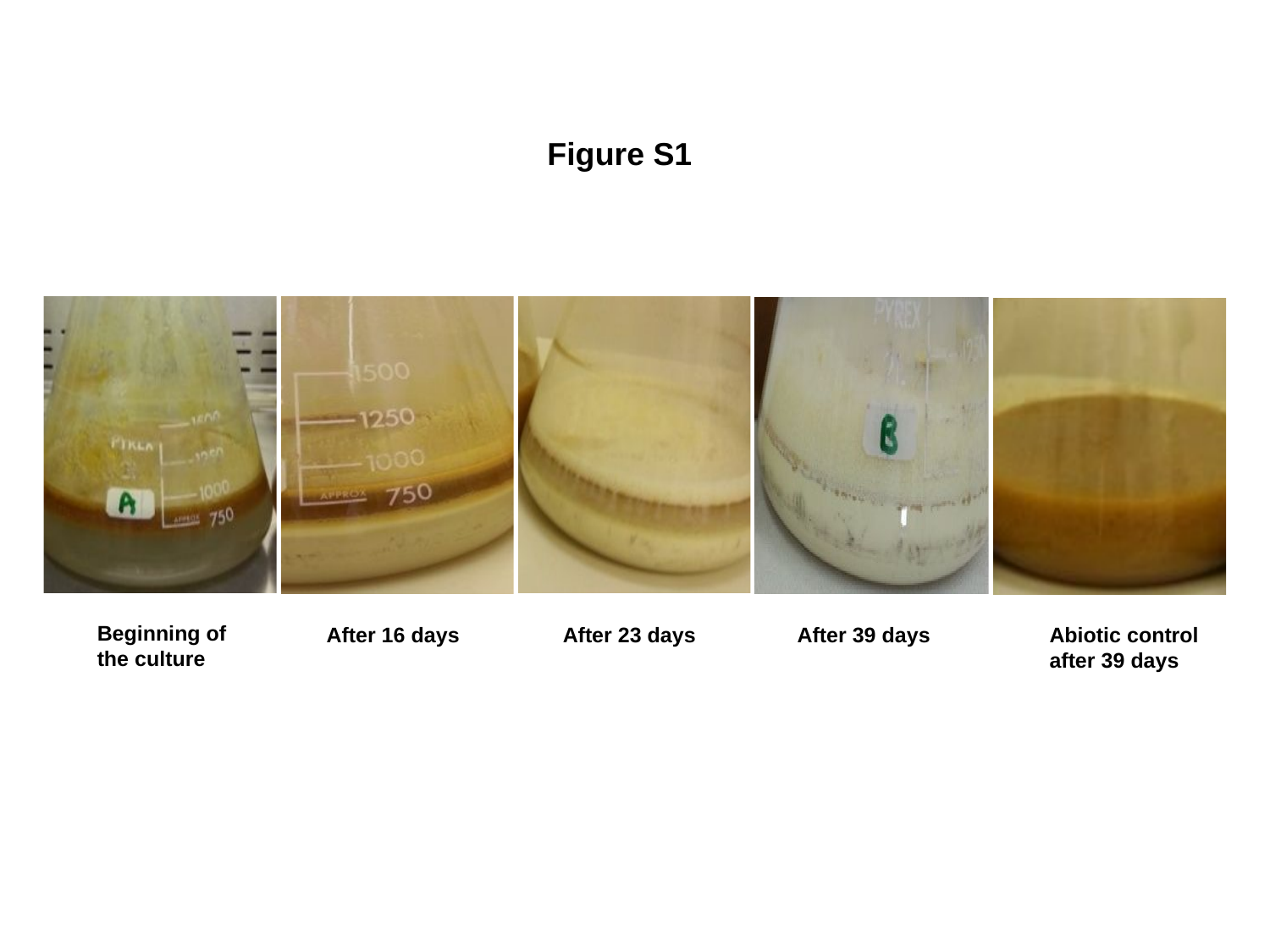

Figure S1
Beginning of the culture
After 23 days
After 39 days
After 16 days
Abiotic control after 39 days
