# Supplementary material for: Simultaneous valorization and biocatalytic upgrading of heavy vacuum gas oil by the biosurfactant‐producing Pseudomonas aeruginosa AK6U
Source: Microb Biotechnol. 2017 Jul 11;10(6):1628–39. doi: 10.1111/1751-7915.12741 (PMC5658591; doi:10.1111/1751-7915.12741)
Supplement: Supplementary file 2 — Fig. S2. Centrifuge bottles showing the cultures of P. aeruginosa AK6U on HVGO (56 days of incubation) after centrifugation at 10 000 rpm for 10 min. [file MBT2-10-1628-s002.ppt]

## Slide 1
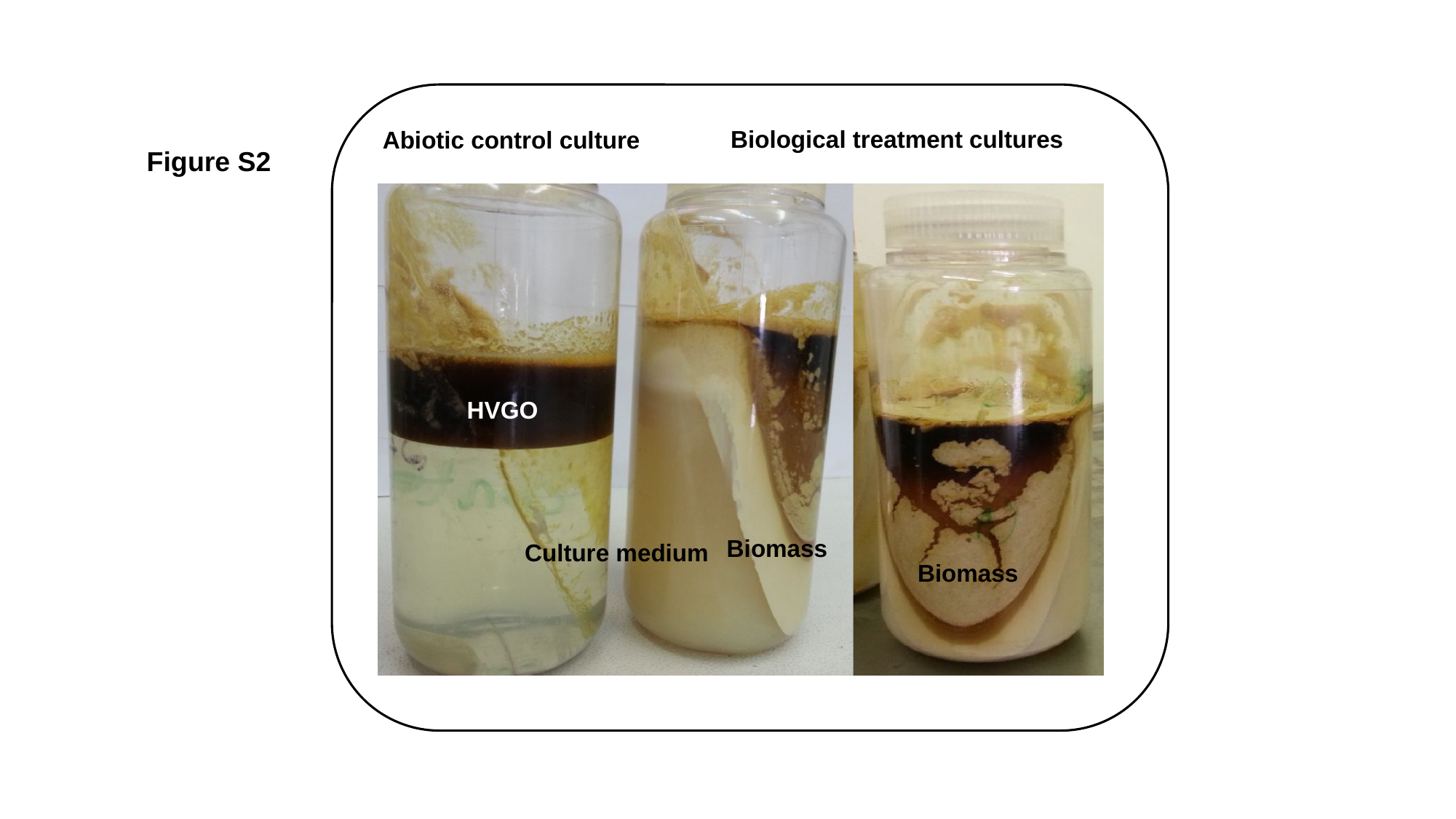

Biological treatment cultures
Abiotic control culture
Figure S2
HVGO
Biomass
Culture medium
Biomass
