# Supplementary figures and images for: Simultaneous valorization and biocatalytic upgrading of heavy vacuum gas oil by the biosurfactant‐producing Pseudomonas aeruginosa AK6U
Source: Microb Biotechnol. 2017 Jul 11;10(6):1628–39. doi: 10.1111/1751-7915.12741 (PMC5658591; doi:10.1111/1751-7915.12741)

## Slide 1
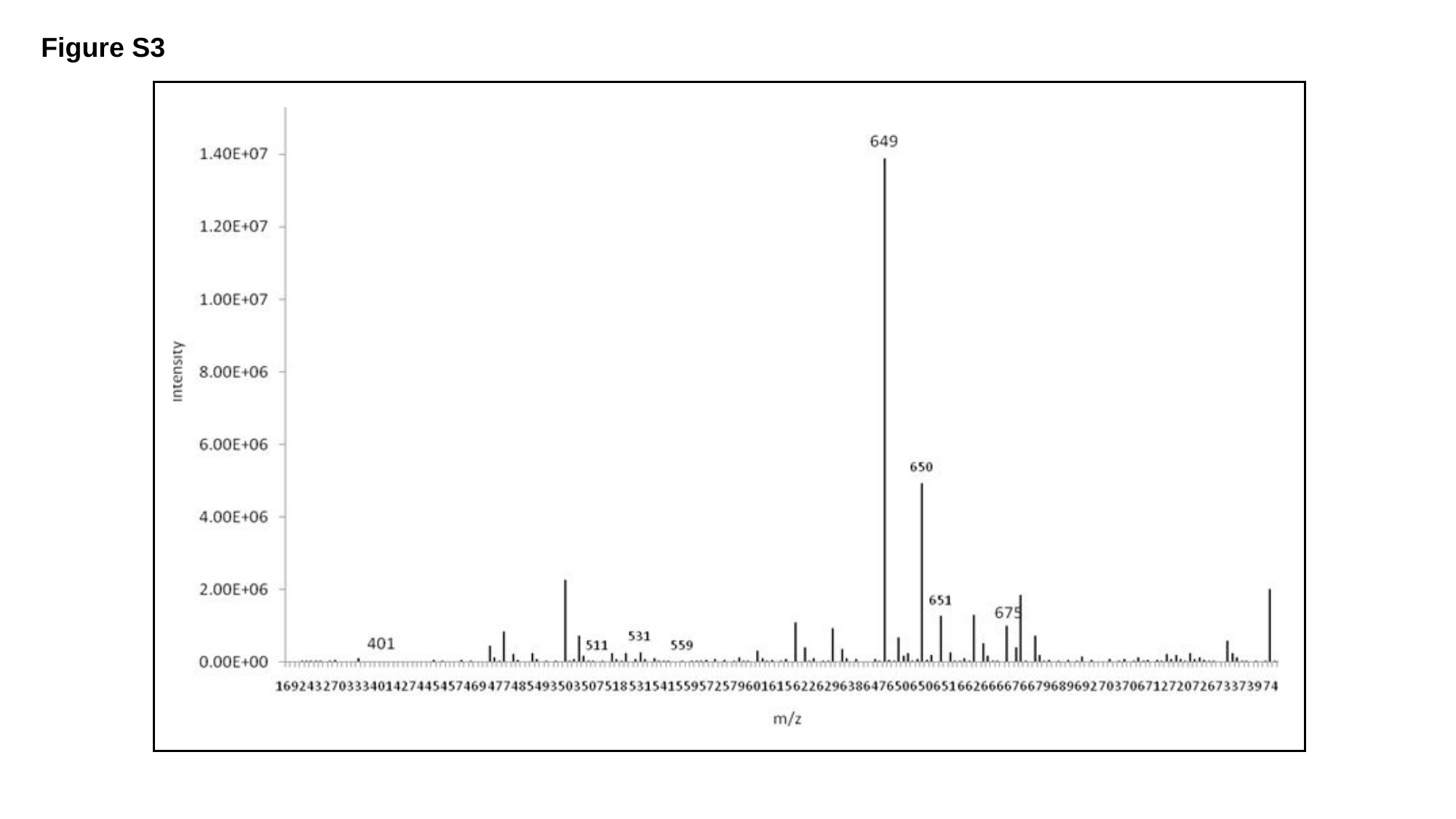

Figure S3

Supplement: Supplementary file 3 — Fig. S3. A mass spectrum of a rhamnolipid produced by P. aeruginosa AK6U grown on HVGO (20% v/v) in mineral salts medium as the sole carbon and sulfur source. [file MBT2-10-1628-s003.pptx]
