# Supplementary material for: Simultaneous valorization and biocatalytic upgrading of heavy vacuum gas oil by the biosurfactant‐producing Pseudomonas aeruginosa AK6U
Source: Microb Biotechnol. 2017 Jul 11;10(6):1628–39. doi: 10.1111/1751-7915.12741 (PMC5658591; doi:10.1111/1751-7915.12741)
Supplement: Supplementary file 4 — Fig. S4. Results of fractional distillation (SimDist) analysis of maltene fractions, extracted from abiotic control and bio‐treated HVGO, into different boiling point fractions (recovered weight %). [Residue = bottom of the barrel fraction with very high BP (> 565 °C), Fuel = heavy viscous fuel oil (BP ≈ 426–565 °C), Diesel fraction (BP ≈ 315–426 °C), Kerosene fraction (BP ≈ 204–315 °C)] [file MBT2-10-1628-s004.pptx]

## Slide 1
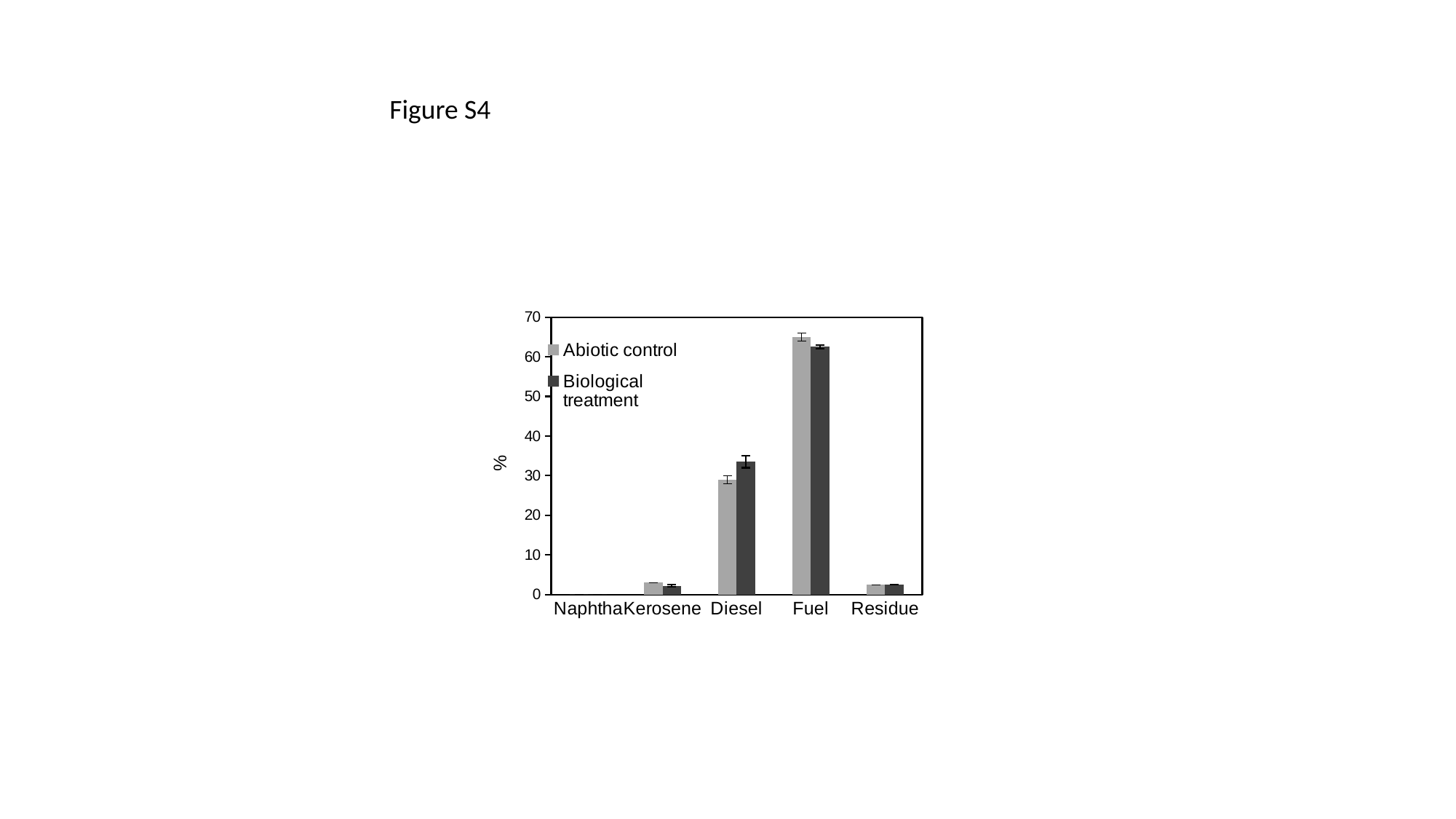

Figure S4
### Chart
| Category | Abiotic control | Biological treatment |
|---|---|---|
| Naphtha | 0.0 | 0.0 |
| Kerosene | 3.0 | 2.25 |
| Diesel | 29.0 | 33.5 |
| Fuel | 65.0 | 62.5 |
| Residue | 2.5 | 2.5 |
